# Association Between Internal Organ/Liver Tumor and External Surface Motion From Cine MR Images on an MRI-Linac

**DOI:** 10.3389/fonc.2022.868076

**Published:** 2022-06-30

**Authors:** Weihua Mao, Joshua Kim, Indrin J. Chetty

**Affiliations:** Department of Radiation Oncology, Henry Ford Health System, Detroit, MI, United States

**Keywords:** motion correlation, MR cine imaging, liver cancer, tumor motion, diaphragm motion, skin motion

## Abstract

**Purposes/Objectives:**

Historically, motion correlation between internal tumor and external surrogates have been based on limited sets of X-ray or magnetic resonance (MR) images. With the recent clinical implementation of MR-guided linear accelerators, a vast quantity of continuous planar real-time MR imaging data is acquired. In this study, information was extracted from MR cine imaging during liver cancer treatments to establish associations between internal tumor/diaphragm and external surface/skin movement.

**Methods and Materials:**

This retrospective study used 305,644 MR image frames acquired over 118 treatment/imaging sessions of the first 23 liver cancer patients treated on an MRI-linac. 9 features were automatically determined on each MR image frame: Lung_Area, the posterior (Dia_Post), dome (Dia_Dome), and anterior (Dia_Ant) points of a diaphragmatic curve and the diaphragm curve point (Dia_Max), the chest (Chest) and the belly (Belly) skin points experiencing the maximum motion ranges; the superior-interior (SI) and posterior-anterior (PA) positions of a target. For every session, correlation analyses were performed twice among the 9 features: 1) over a breath-hold (BH) set and 2) on a pseudo free-breathing (PFB) generated by removing breath-holding frames.

**Results:**

303,123 frames of images were successfully analyzed. For BH set analysis, correlation coefficients were as follows: 0.94 ± 0.07 between any two features among Dia_Post, Dia_Dome, Dia_Max, and Lung_Area; 0.95 ± 0.06 between SI and any feature among Dia_Post, Dia_Dome, Dia_Max, or Lung_Area; 0.76 ± 0.29 between SI and Belly (with 50% of correlations ≥ 0.87). The PFB set had 142,862 frames of images. For this set, correlation coefficients were 0.96 ± 0.06 between any two features among Dia_Post, Dia_Dome, Dia_Max, and Lung_Area; 0.95 ± 0.06 between SI and any feature among Dia_Post, Dia_Dome, Dia_Max, or Lung_Area; 0.80 ± 0.26 between SI and Belly (with 50% of correlations ≥ 0.91).

**Conclusion:**

Diaphragmatic motion as assessed by cine MR imaging is highly correlated with liver tumor motion. Belly vertical motion is highly correlated with liver tumor longitudinal motion in approximately half of the cases. More detailed analyses of those cases displaying weak correlations are in progress.

## Introduction

Internal tumor motion due to respiration compromises the precision of radiation therapy and efficacy at delivering high radiation doses to control the tumor while minimizing side effects to adjacent normal tissues. Large margins have been added to expand the clinical target volume to a much larger planning treatment volume (PTV) (1–3). It is vital to locate tumors and critical structures in real-time to minimize irradiation of normal tissue (4–7). However, it is difficult to directly track internal organ motions in real-time by common radiation therapy equipment. X-ray based fluoroscopy imaging delivers too much extra imaging dose with limited soft tissue contrast (8). Implanted fiducial markers only provide locations of limited points with the risk of side effects from fiducial implantation and marker migration (9). Intensive studies have been reported to identify the correlation between internal tumor motion and external surrogates (10–23). Correlations between internal tumor motions and external surrogates have been based on computed tomography (CT) or magnetic resonance (MR) volumetric image sets or dynamically updated with intermittent X-ray or MR images during treatment. All these studies were performed based on limited patient data. Previous reports that focused on cine MR images to define correlations between different surrogates have used MR images acquired over a limited time period and outside the context of actual radiation therapy treatment delivery. For example, Paganelli et al. reported correlation studies between internal features and external surrogates based on 120 frames of MR cine images over 74.4 seconds per patient (24); Yang et al. reported correlation between diaphragm and liver tumor based on MR images within 15~30 seconds (25). It is clinically important to study the correlation over a time period covering an entire radiation therapy treatment from positioning patient to completing dose delivery, which lasts for at least 10 minutes. Moreover, breathing patterns acquired at simulation can often be different than those at time of actual treatment. With the recent clinical implementation of MR-guided linear accelerators, a vast quantity of continuous planar real-time MR imaging data is acquired at 4 frames/second as part of the radiation therapy treatment delivery process (5–7). In the first 9-months after installation of an MR-guided linear accelerator at our institution, 23 liver cancer patients had been treated by stereotactic body radiation therapy (SBRT). MR cine images were acquired for about one hour per patient. In this study, liver tumor motion data was extracted from the treatment MR cine imaging data to establish associations between internal tumor/diaphragm and external surface/skin movement from the same image sets for the entire treatment session. This would be the first report that includes a large quantity of cine images acquired during actual treatment for the full treatment session. This is very different from reports in the literature, which were based on patient data over a short period of time. With such an enormous amount of data, patient respiratory diversity and variation were represented by the changing respiratory pattern over the course of a full treatment. This is different from irregular breathing usually observed during 4D CT scans, which might be due to irregular respiration amplitude.

## Methods and Materials

This retrospective study used patient cine images acquired during routine stereotactic body radiation therapy (SBRT) treatments on a low field (0.35 T) MRI-linac (MRIdian, ViewRay, Mountain View, CA). 23 liver cancer patients (listed in **Table 1**) were enrolled in this Internal Review Board (IRB)-approved (IRB #12934) study. All patients were prescribed to 50 Gy in 5 fractions. In certain cases, one planned fraction of treatment might be interrupted and the remainder of this fraction of treatment would be resumed later. In that case, one planned fraction of treatment could span multiple sessions. 305,644 frames of MR sagittal images were acquired as a part of the radiation therapy process.

**Table 1:** List of patients with planning target volume (PTV) size and average longitudinal and lateral locations.

Patients	Gender	Age	Diagnosis	PTV (cm^3^)	Longitudinal location (mm)	Lateral location
LV01	F	89	Secondary malignant neoplasm of bone	40	40	0.60
LV02	M	71	Liver cell carcinoma	148	69	0.70
LV03	M	85	Secondary malignant neoplasm of liver and intrahepatic bile duct	50	106	0.45
LV04	F	54	Liver cell carcinoma	46	61	0.58
LV05	M	65	Liver cell carcinoma	181	49	0.66
LV06	M	68	Liver cell carcinoma	71	69	0.47
LV07	M	72	Liver cell carcinoma	937	71	0.68
LV08	M	89	Liver cell carcinoma	24	39	0.53
LV09	F	90	Intrahepatic bile duct carcinoma	111	83	0.72
LV10	M	56	Liver cell carcinoma	63	57	0.69
LV11	M	71	Secondary malignant neoplasm of liver and intrahepatic bile duct	118	61	0.55
LV12	M	78	Liver cell carcinoma	12	108	0.52
LV13	M	88	Intrahepatic bile duct carcinoma	121	49	0.55
LV14	M	68	Liver cell carcinoma	253	84	0.57
LV15	M	68	Liver cell carcinoma	48	89	0.31
LV16	F	86	Secondary malignant neoplasm of liver and intrahepatic bile duct	29	14	0.55
LV17	M	83	Secondary malignant neoplasm of liver and intrahepatic bile duct	231	95	0.78
LV18	M	68	Secondary malignant neoplasm of liver and intrahepatic bile duct	48	51	0.53
LV19	F	65	Liver cell carcinoma	65	20	0.53
LV20	M	78	Intrahepatic bile duct carcinoma	59	61	0.57
LV21	F	70	Intrahepatic bile duct carcinoma	281	27	0.75
LV22	F	76	Liver cell carcinoma	115	57	0.49
LV23	M	71	Liver cell carcinoma	60	82	0.48

Longitudinal location is the longitudinal distance between the target center and diaphragmatic dome. Lateral location is the lateral off-center ratio, which is the lateral distance between target center and spine as a ratio of the lateral distance between inner edge of the thoracic cage and spine.

**Figure 1:**
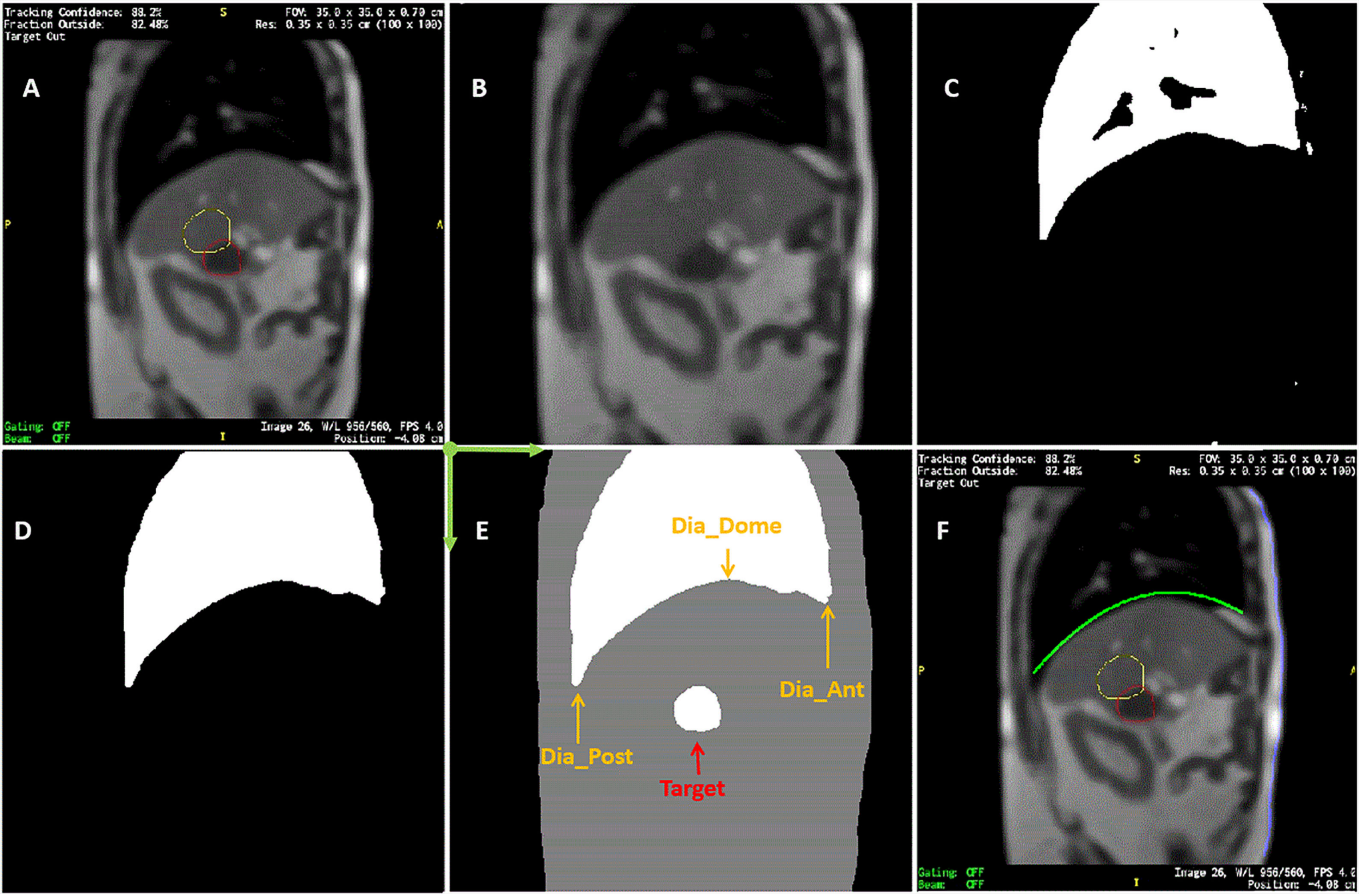
Sample of MR image detection. **(A)** Acquired image. Yellow line contoured the expected target boundary and red line contoured the detected target. Every MR image frame had blank frame borders with information texts attached to four sides of MR images. **(B)** Cropped image after contours were removed. **(C)** Initially detected lung based on intensity threshold. **(D)** refined lung structure. **(E)** Detected body, lung, and target contours superimposed. Dia_Dome, Dia_Post, Dia_Ant, are Target are labeled. Coordinate system origin locates at the upper-left corner and axis directions were illustrated in green arrows. **(F)** Detected body and diaphragmatic curve superimposed back to the original image.

After 3-dimensional (3D) MRI images were acquired to setup patients for treatment, this MRI-linac continuously acquired four sagittal MR image frames per second during treatment. Dimensions of each frame were 100 x 100 and pixel sizes were 3.5 mm x 3.5 mm. After a patient was positioned for treatment, an initial target tracking structure was manually contoured on the initial volumetric image set and a tracking boundary structure was automatically generated as an isotropic 3 mm expansion of the tracking structure. The treatment software routinely monitored target motion. It deformably propagated the target tracking structure automatically onto each newly acquired image frame. Additionally, the tracking boundary structure was statically copied onto each image frame. The treatment software continuously monitored whether the detected target was within this boundary. Treatment beam delivery would be held whenever the target was out of the boundary by a preset percentage (5% for liver SBRT treatment) and the beam delivery would resume when the target moved back in the boundary. The tracking structure may be different from the target but must represent target motion. In this article, we regard tracking structures as targets. Targets were mapped as red contours and target boundaries were mapped as yellow contours (as shown in **Figure 1A**). They were overlaid on grayscale MR images. Cine images were saved and exported as videos for each treatment session. The videos have dimensions of 512 x 512 and pixel sizes are 0.79 mm x 0.79 mm. In-house software was developed to analyze images in Matlab (MathWorks, Natic, MA) following the below steps:

1. Read video files and load each frame of MR images.
2. Crop images to remove embedded borders and keep MR images only.
3. Detect the contours of targets and target boundaries. Fill target structures and calculate the center of mass in Posterior-Anterior direction (PA) and Superior-Inferior direction (SI). PA and SI will be used throughout as referring to the target positions in two directions. Remove contours and fill blank pixels with the average of surrounding pixels, as shown in **Figure 1B**.
4. Use an intensity threshold to detect the body contour on the image.
5. Use an intensity threshold in the body to detect lungs as shown in **Figure 1C**. Matlab functions including hole filling, eroding, expanding, and selection of the largest areas were used to refine lung detection as shown in **Figure 1D**. Parabolic curves were used to fit diaphragmatic curve (upper portions). **Figure 1E** superimposed the lung structures and filled target onto the body contour.
6. Parabolic curve fitting results and anterior body contours were mapped back to the original images as a record (**Figure 1F**).

On each frame of the cine MR treatment acquisition, in addition to target positions PA and SI, our Matlab program automatically determined the following four lung area features as illustrated in **Figure 1E** (26): (1) Lung_Area - the total area of the detected lung; (2) Dia_Post - the most posterior point on the patient diaphragmatic curve; (3) Dia_Dome - the dome or apex of the diaphragmatic curve after curve-fitting; (4) Dia_Ant - the most anterior point of the patient diaphragmatic curve. Three more features were determined after analyzing motion of each session. The point on the diaphragm that experienced the maximum range of longitudinal motion over a treatment session was selected, and its longitudinal positions were extracted as Dia_Max. The average Dia_Ant point per session was used to separate chest and belly regions on the anterior body surface. The chest skin point and the belly skin point that experienced the maximum range of vertical motion at the chest and belly region, respectively, were selected. Their vertical positions are Chest and Belly. Typically, respiratory motion is usually estimated using external surrogates such as the vertical motion of surface points on the upper abdomen. For example, the Varian Real-time Position Management (RPM) system uses an infrared camera to track the vertical motion of a block placed on the patient’s anterior upper abdominal skin surface while the Philips bellows belt system is placed around the patient’s belly to measure pressure changes due to respiration. We searched for the skin point with maximum motion range to simulate the optimal external surrogate.

A pre-analysis screening process was automatically carried out for every session. There were certain situations when the target tracking structure was not tracked correctly due to sudden large target excursions. Therefore, a pre-analysis screening process was automatically carried out for every session. Histograms with a bin width of 3.5 mm (the pixel size of original images) were generated for both target positions (SI and PA) and the Belly position. A cutoff frequency was defined as 1% of the maximum frequency, which was used to determine cutoff position thresholds on the upper and lower sides of the target position that occurred most frequently on the histogram. Frames falling outside of the cutoff positions were regarded as outliers (extreme and isolated positions), and they were excluded from correlation analysis.

**Figure 2:**
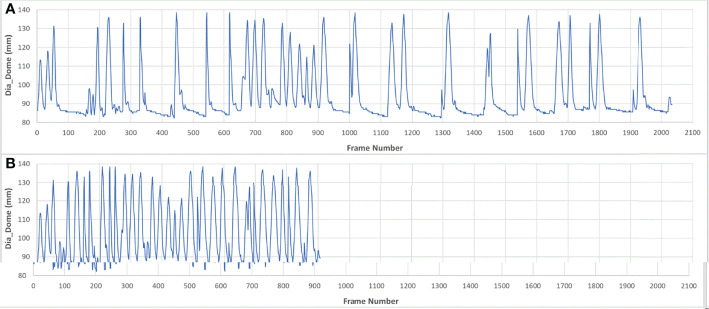
Sample of breath-hold (BH) data set **(A)** and its pseudo free-breathing (PFB) set **(B)**.

Pearson correlation coefficients were calculated twice for each pair of features among the 9 features described above for every session. The first correlation analysis was computed over the full set of image frames, which was designated as the breath-hold (BH) set. The MR images were acquired during breath-hold treatment and about half of the image frames were acquired while patients were holding their breath. To eliminate the adverse effects of such unbalanced distribution of motion positions, the second analysis was performed on pseudo free-breathing (PFB) data sets, which were generated by removing image frames identified as being at breath-holding from the BH set. The analysis program automatically compared the Dia_Dome position of each frame with the Dia_Dome positions of the 3 immediately preceding and following frames. If the Dia_Dome range was less than one pixel (3.5 mm), this frame would be regarded as a breath-holding frame. **Figure 2** illustrated a BH set of images and its PFB set. Statistical analyses of comparisons between BH and PFB set analysis results were performed using Student’s *t*-test (1 tail, at the alpha = 0.05 significance level).

For every session, the sagittal plane to be tracked during cine imaging was selected using the initial volumetric MRI image. The lateral position of the selected sagittal plane was recorded for each session. The lateral positions of the target center, spine, and inner edge of the thoracic cage were manually detected using an axial slice of the initial volumetric MRI that was located at the center of the tracking target. Lateral off-center ratio was calculated as the lateral distance between target center and spine divided by the distance between lateral thoracic cage edge and spine. The smaller the ratio, the closer to the body midline. Average lateral off-center ratios were listed in **Table 1**.

## Results

**Figure 3:**
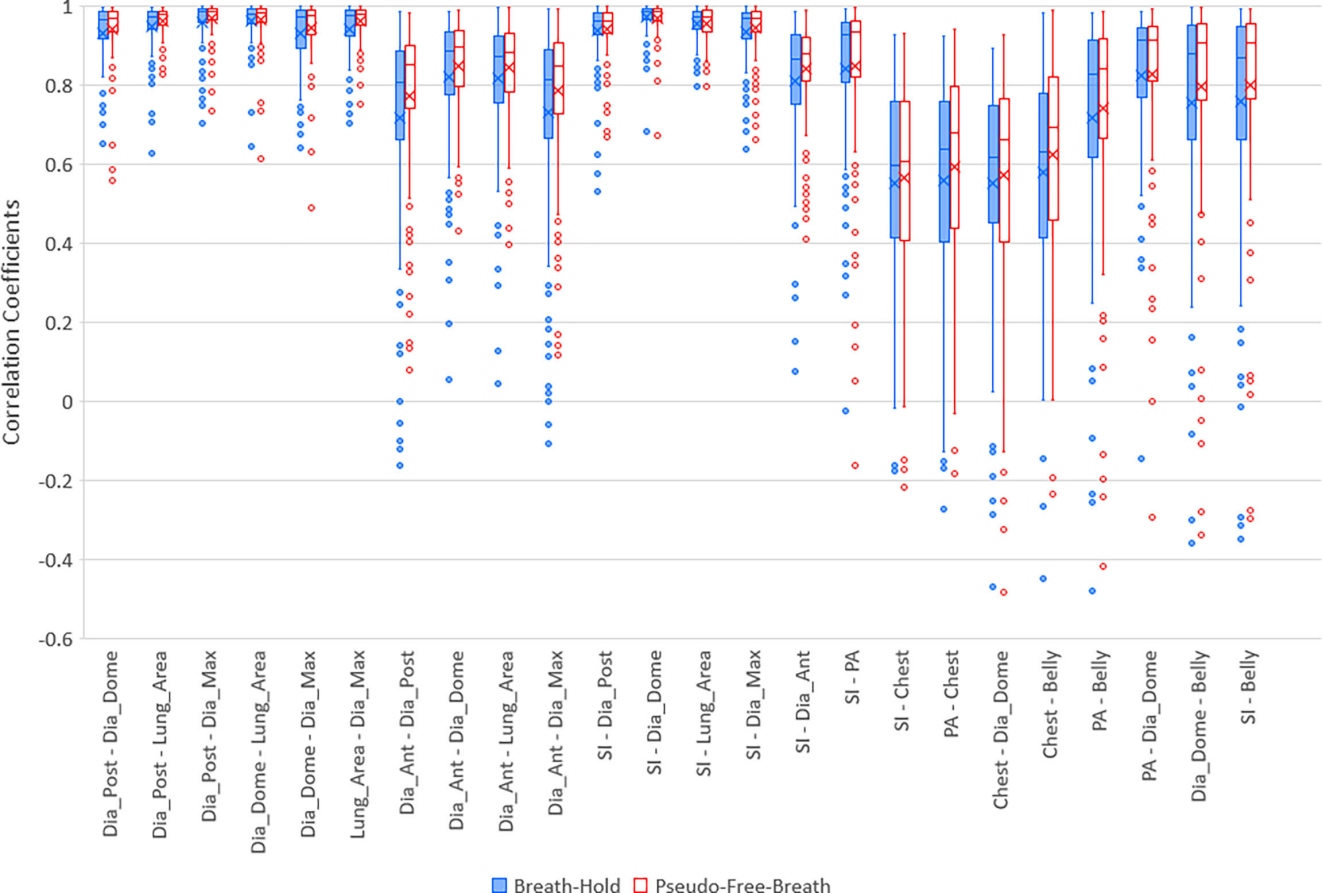
Boxplots of selected correlation coefficients between two of 9 parameters, PA, SI, Dia_Post, Dia_Dome, Dia_Ant, Lung_Area, Dia_Max, Chest, and Belly for Breath-Hold and Pseudo-Free-Breath. Median and Average correlation coefficients are marked by horizontal line and “x” in each box, respectively. Interquartile range (IQR) is the difference between the 3^rd^ quartile (Q3, the upper side of each box) and the 1^st^ quartile (Q1, the lower side of each box). The upper whisker ends at maximum correlation coefficients while bottom whisker ends at Q1 – 1.5 * IQR. Any correlation coefficients smaller than Q1 – 1.5 * IQR are considered to be outliers and are displayed represented by dots.

303,123 frames of 2D MR images were analyzed in the BH set over 118 imaging sessions for 23 patients. Less than 1% of images were excluded where the target was not correctly detected by the system. The PFB set analysis used 142,862 of the image frames. **Figure 3** illustrates selected correlation coefficients of 36 feature pairs among the 9 features defined above for BH and PFB analyses. **Figure 3** shows that correlation results of the PFB set are slightly better than BH set analysis results. Analysis was done to compare correlation coefficients per session. The PFB set showed slightly better correlation coefficients among diaphragmatic features (differences of 0.01 ± 0.04 with p-value < 0.01) and similar correlation coefficients between SI and any one of the four diaphragmatic features (differences of 0.00 ± 0.03) while the analysis using PFB set showed better correlation coefficients between Belly and SI (differences of 0.04 ± 0.08 with p-value < 0.01). This shows that excessive breath-holding image frames at end exhalation would affect correlation analysis and the PFB results should be used for the free breathing scenario.

Five lung/diaphragmatic features behave differently, and it is essential to focus on the feature with the best correlation to external motion. High aggregate cross correlations occur between any two features among Dia_Post, Dia_Dome, Dia_Max, and Lung Area, as the left 6 groups of boxplots in **Figure 3** demonstrate. They are 0.94 ± 0.07 and 0.96 ± 0.06 for BH and PFB set analyses, respectively. However, the fifth feature, Dia_Ant, did not have high aggregate correlations with other features. They are 0.77 ± 0.23 and 0.81 ± 0.17 for BH and PFB set analyses, respectively. After reviewing the Dia_Ant results, we found a complex intensity scheme. This may be due to the proximity of the diaphragm to bones as well as possible effects of ascites on the precision of identifying the proper Dia_Ant position. Among the four features, Dia_Dome results achieved the highest correlation with SI. This suggests that Dia_Dome is the best landmark to represent diaphragmatic motions, which is consistent with findings reported by Yang et al. (25). It should be noted that the superior regions of the lungs may sometimes be outside of the field of view. Therefore, the Lung_Area parameter may not represent the full lung area which may degrade the accuracy of the correlation between Lung_Area and other features.

**Figure 4:**
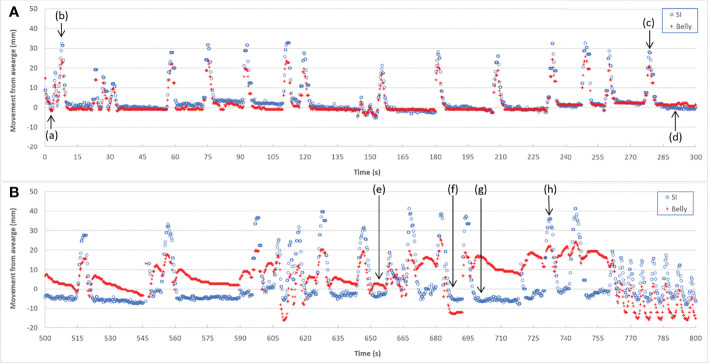
Target SI and Belly motion as functions of time for Session #3 **(A)** and Session #4 **(B)** of patient LV13.

**Figure 5:**
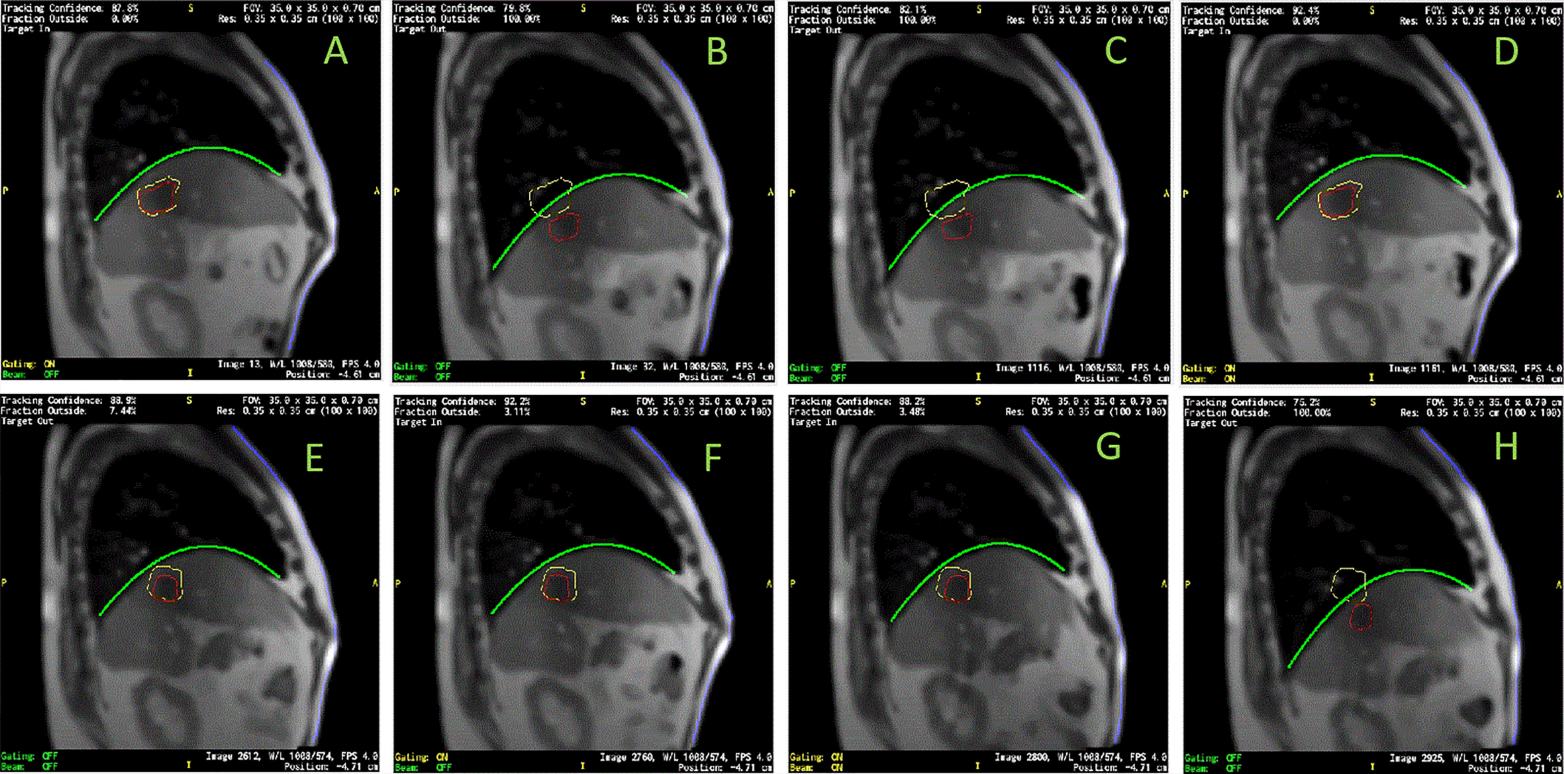
Images selected from two sessions of patient LV13. **(A–D)** were from Session #3 and **(E–H)** were from Session #4 as labeled in **Figure 4**. Target/tracking structures are in red; boundaries of tracking structure are in yellow; curve-fitting results of diaphragmatic curves are in green; anterior skin extracted from body contours are in blue. Frames **(A–H)** indicate the frames (a-h) labeled in **Figure 4**.

**Figure 6:**
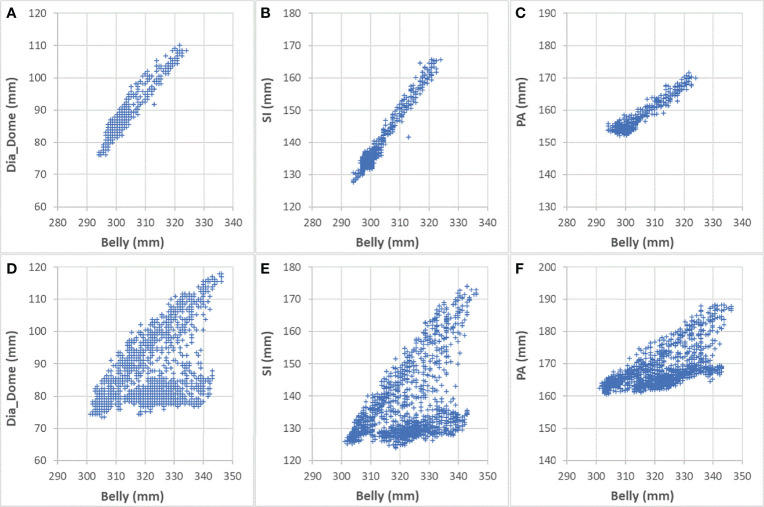
Comparing correlations with Belly for Sessions #3 and #4 of patient LV13. **(A)** Dia_Dome as a function of Belly in Session #3; **(B)** SI as a function of Belly in Session #3; **(C)** PA as a function of Belly in Session #3; **(D)** Dia_Dome as a function of Belly in Session #4; **(E)** SI as a function of Belly in Session #4; **(F)** PA as a function of Belly in Session #4.

**Figure 7:**
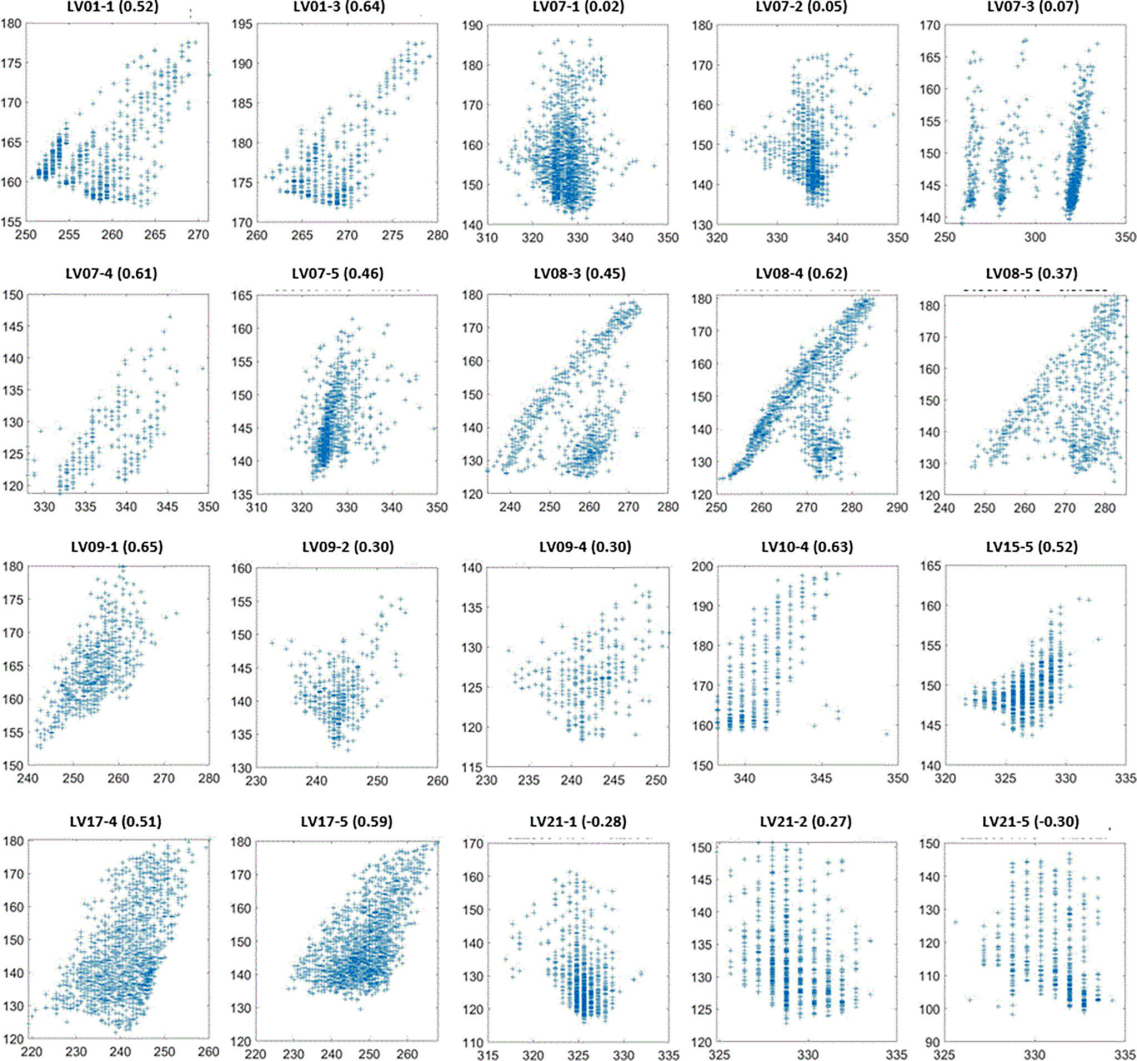
SI (vertical axes in mm) as a function of Belly (horizontal axes in mm) for the sessions with the lowest 20 SI-Belly correlation coefficients. Figure titles include patient ID and Session #. Correlation coefficients are in parentheses.

High correlation coefficients (0.95 ± 0.06) occurred as combined result of all feature pairs between SI and any of Dia_Post, Dia_Dome, Dia_Max, and Lung_Area for either BH or PFB set analyses. SI-Belly Correlation coefficients are 0.76 ± 0.29 (with 50% of correlations ≥ 0.86) and 0.80 ± 0.26 (with 50% of correlations ≥ 0.91) for BH and PFB set analyses, respectively. SI-Belly correlation is patient dependent and may vary in different sessions as summarized in **Table 2**. To verify and further study SI-Belly correlation variations, motion details were compared. Particularly, patient LV13 SI-Belly correlation coefficients varied between 0.65 and 0.98 among four sessions. **Figure 4** illustrates portions of SI and Belly results as functions of time of the same patient, LV13, over Sessions #3 and #4. **Figure 5** shows four frames selected from the two sessions of patient LV13 displayed in **Figure 4**. **Figure 6** compares correlations with Belly between the two sessions. These two sessions showed very different internal-external motion correlations. Furthermore, **Figure 7** illustrates SI-Belly correlations for the sessions with the lowest 20 correlation coefficients.

**Table 2:** List of average, standard deviation (Stdev), minimum, and maximum correlation coefficients between SI and Belly for each patient.

Patient	Number of Sessions	Average	Stdev	Minimum	Maximum	Stdev/Average	Range/Average/2
LV01	5	0.75	0.17	0.52	0.95	0.22	0.29
LV02	6	0.89	0.05	0.81	0.95	0.06	0.08
LV03	5	0.94	0.03	0.90	0.97	0.03	0.04
LV04	6	0.93	0.09	0.76	0.98	0.09	0.12
LV05	6	0.84	0.07	0.74	0.89	0.09	0.09
LV06	5	0.85	0.05	0.77	0.91	0.06	0.08
LV07	5	0.24	0.27	0.02	0.61	1.14	1.23
LV08	5	0.63	0.23	0.37	0.88	0.36	0.40
LV09	5	0.56	0.23	0.30	0.78	0.42	0.43
LV10	5	0.73	0.12	0.63	0.92	0.17	0.20
LV11	5	0.98	0.01	0.98	0.99	0.01	0.01
LV12	5	0.88	0.06	0.78	0.93	0.07	0.08
LV13	4	0.89	0.16	0.65	0.98	0.18	0.19
LV14	6	0.95	0.04	0.90	0.98	0.04	0.05
LV15	7	0.85	0.16	0.52	0.97	0.19	0.27
LV16	5	0.96	0.03	0.91	0.98	0.03	0.04
LV17	5	0.65	0.10	0.51	0.77	0.15	0.20
LV18	5	0.90	0.06	0.81	0.96	0.07	0.09
LV19	5	0.93	0.01	0.92	0.94	0.01	0.01
LV20	5	0.94	0.02	0.93	0.97	0.02	0.02
LV21	5	0.18	0.63	-0.30	0.87	3.56	3.31
LV22	5	0.93	0.05	0.83	0.96	0.06	0.06
LV23	3	0.91	0.07	0.83	0.97	0.08	0.07

Patients with the most linear correlations were highlighted in green. Patients with the least linear correlations were highlighted in yellow.

## Discussion

It should be noted that although PFB results show better internal-external correlations, both PFB and BH are different from natural free breathing. When patients were asked to hold their breath at the end of exhale, their lung volumes were generally smaller than those at the end of exhale during a free breathing cycle. Immediately after stressful breath-holds, patients might breathe faster than during free breathing. One limitation of this study is that all cine images available to authors were from breath-hold treatments. The conclusions are valid for breath-hold treatment. In addition, if a patient always breathes with the same respiratory pattern, the internal-external motion correlation should be identical for PFB and free-breathing. During PFB the patient may experience a slightly larger motion range due to deep breath-hold relative to free breathing. In this case, PFB results should be identical to free-breathing results. However, stressful breath-holds might trigger different respiratory patterns, which could alter the internal-external correlations.

Both Chest and Belly features represent external surrogates but behave very differently. Chest has weaker correlation in aggregate than any other feature. If a patient only performs chest breathing, Dia_Dome and SI would move linearly with Chest while minimal motion of the Belly would be expected. However, observed correlation coefficients between Chest and Dia_Dome were 0.55 ± 0.27 and 0.57 ± 0.28 for BH and PFB set analyses, respectively. Correlation coefficients between Chest and SI were only 0.55 ± 0.26 and 0.57 ± 0.25 for BH and PFB set analyses, respectively. This indicates that no patient experienced pure chest breathing, and the position of the chest during chest breathing is not always a good surrogate for the breathing pattern. Another reason could be that Chest experienced the smallest range of motion among the 9 features investigated. Motion ranges were 16 ± 6 mm, 19 ± 8 mm, and 25 ± 11 mm for Chest, PA, and Belly, respectively. Small motion ranges might result in larger uncertainties, especially when the pixel size of the original MR images was relatively large (3.5 mm). Belly motion showed improved correlation with both diaphragmatic and target motions compared to Chest motion. Correlation coefficients between Belly and Dia_Dome are 0.76 ± 0.30 and 0.80 ± 0.28 for BH and PFB set analyses, respectively.

One of our goals was to determine the correlation between Belly and target motion. The correlation coefficient between Belly and SI was 0.76 ± 0.29 using the BH set. Half of the imaging sessions had correlation coefficients between Belly and SI greater than or equal to 0.87. As listed in **Table 2**, 9 patients, highlighted in green, have linear correlation between SI and Belly (with minimum correlation coefficients ≥ 0.81) in every imaging session. Their inter-session correlation coefficient variations are presented by ratio of standard deviation over average (0.06 or less) and ratio of half range over average (0.09 or less) as listed in **Table 2**. **Table 2** shows that more than half (12/23) of the patients have average correlation coefficients greater than 0.88 in their imaging sessions. The remaining patients have average correlation coefficients less than 0.86. As highlighted in yellow in **Table 2**, five patients had weak SI-Belly correlations (with maximum correlation coefficients ≤ 0.88) in every imaging session. If these data were excluded as outliers, PFB correlation coefficients between SI and Belly would be 0.89 ± 0.10 for the 93 sessions of 18 patients. Except for the nine patients with high linear SI-Belly correlations (highlighted in green in **Table 2**), 15 patients changed their respiratory patterns in at least one imaging session. As an example, **Figures 4**–**6** compare Belly and SI motions of patient LV13 over two different sessions. The PFB correlation coefficients were 0.98 and 0.65 for Sessions #3 and #4, respectively. Compared with **Figures 5A** and **D or B** and **C, E–G** shows that the Belly moved while the diaphragm (Dia_Dome) and target (SI) position remained stationary, causing a wide spread of data points along the Belly position axis at breath-holding positions. The position of each figure along the breathing trace were illustrated in **Figure 4B**. This means that the baseline Belly position varies over a period of just 60 seconds. In the same session, the right portion of the breathing trace displayed in **Figure 4B** plots a period of greater than 30 seconds when the target moves over a range of 25 mm while the Belly moved correspondingly but at a very different Belly baseline. More specifically, **Figure 4B** illustrated a repeating trend that at breath hold, Belly (red “+”) tended to gradually drift along the posterior direction while SI (blue “o”) and diaphragm remained stationary. This is a potential limitation of breath-hold studies. Patients might change their breathing patterns relative to their normal free breathing pattern. This deviation from normal breathing pattern more likely happened at breath-holding phases. The PFB set was created from the full image set by removing breath-holding frames based on diaphragm positions and some frames with belly drifting were removed. This resulted in a better SI-Belly correlation relative to the full BH set analysis. It should be noted that Pt LV21 had the least linear correlation between SI and Belly as shown in **Figure 7**. Video review found that this patient’s belly was located on the border of the MR imaging field of view (FOV) due to patient size, and a portion of the belly kept moving in and out of imaging FOV during treatment. As a result, the extracted Belly results won’t represent the optimal external motion.

**Figure 8:**
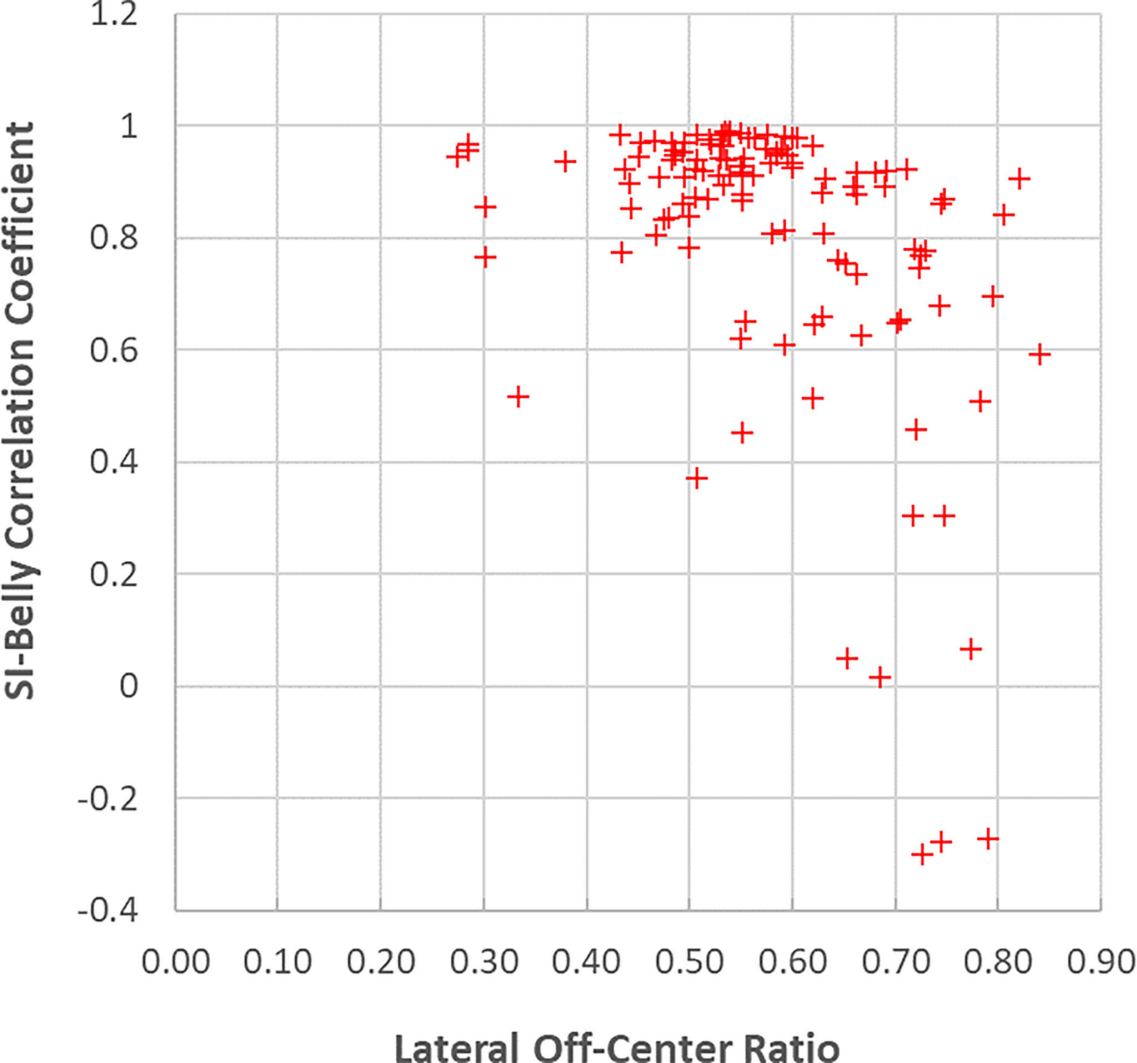
SI-Belly correlation coefficients as a function of the lateral off-center ratio.

This study utilized over 300,000 frames of images, which recorded respiratory information, including both internal tumor/organs and external skin motions, lasting for an average of 54 minutes per patient over different days. For lung inhalation, chest breathing may expand the thoracic cage only while abdominal breathing engages the belly and abdomen. A combination of chest and abdominal breathing or even more complicated breathing patterns may occur during radiation therapy treatment. For example, chest breathing may be performed while the belly/abdomen holds at different levels. Once the respiratory pattern changed, the relationship between SI and Belly would cease to be linear. **Figure 7** indicates that several sessions have multiple breathing patterns. This implies that an internal-external correlation model cannot be fully determined during a single session. It is unclear how or when a patient may change his/her respiratory pattern. The respiratory pattern may be interrupted by breath holding, particularly when patients were frequently asked to adjust the breath hold level to ensure that the target remains inside the target boundary. On the other hand, patient body movement, such as body rolling, might result in additional belly shift on the sagittal MR cine images, which contributes to respiratory pattern changes. This should be more likely to happen when the sagittal imaging plane is located farther away from the body midline, where the lateral skin slope becomes larger. The SI-Belly correlation coefficient is illustrated as a function of the lateral off-center ratio in **Figure 8**. This indicates that the dispersion of SI-Belly correlation coefficients increases with the lateral off-center ratio. Further studies should involve 3-dimensional patient movement. Later treatment software versions allow for acquiring cine images on multiple planes, which would help to eliminate this kind of uncertainty.

A hysteresis relationship or phase shift between internal (SI) and external (Belly) features has been suggested by many reports (2, 16, 17, 20, 23, 27). However, this was not observed from the data acquired in this study. **Figure 7** illustrates the 20 sessions with the lowest SI-Belly correlation coefficients. The non-linear relationship between SI and Belly could not be explained by a hysteresis loop. As illustrated in **Figures 4**–**6**, Pt LV13 changed respiratory patterns within a single session. **Figures 4E–G** show that the abdomen was changing position even while the target (red contour) remained stationary (within the boundary in yellow). Generally, when a patient exhales during abdominal respiration, the diaphragm moves superiorly and the belly moves posteriorly, which leads to a linear relationship between SI and Belly. However, the patient may hold either with the belly fully (**Figure 4G**) or partially (**Figure 4E**) expanded, which leads to uncertainty in the Belly position at a given breathing phase. As illustrated in **Figure 7**, the sessions with the lowest SI-Belly correlation coefficients demonstrated that even when looking at a single phase (end inhalation), the Belly position would migrate over the course of treatment, which is shown as a wide spread of Belly positions at superior SI locations during holding-breath. This could not be explained by hysteresis.

The linear relationship between diaphragm and target longitudinal motion was confirmed. Correlation coefficients between SI and Dia_Dome were 0.97 ± 0.04 for either BH or PFB set analyses. This is reasonable since the target is underneath the diaphragmatic curve. Although this study is based on liver cancer radiation therapy, the correlation between target and internal diaphragmatic motion could be extended to lung tumor motions. In addition to MR imaging, ultrasound may be used to monitor diaphragmatic motion non-invasively. This also suggests that tracking diaphragmatic motions using an ultrasound technique could be used to monitor liver or lung tumor motions efficiently (28). Chest and Belly motion can be monitored by external surrogates, such as Varian RPM, Philips Bellows, or optical surface imaging systems.

## Conclusions

Diaphragmatic motion as assessed by cine imaging on an MRI-linac is highly correlated with liver tumor motion, and the diaphragmatic dome could be a good indicator of liver tumor motion. Care should be taken when using an external skin motion surrogate positioned at the belly, since this surrogate only has linear correlation with liver tumor longitudinal motion in approximately half of the cases. More detailed analyses of those cases displaying weak correlations are under further investigation.

## Data Availability Statement

The raw data supporting the conclusions of this article will be made available by the authors, without undue reservation.

## Ethics Statement

The studies involving human participants were reviewed and approved by Henry Ford Health System Internal Review Board. Written informed consent for participation was not required for this study in accordance with the national legislation and the institutional requirements.

## Author Contributions

WM was responsible for the research design, data collection and analysis, and manuscript preparation. JK and IC participated into the research design and manuscript preparation. All authors contributed to the article and approved the submitted version.

## Conflict of Interest

The authors declare that the research was conducted in the absence of any commercial or financial relationships that could be construed as a potential conflict of interest.

## Publisher’s Note

All claims expressed in this article are solely those of the authors and do not necessarily represent those of their affiliated organizations, or those of the publisher, the editors and the reviewers. Any product that may be evaluated in this article, or claim that may be made by its manufacturer, is not guaranteed or endorsed by the publisher.
